# Impedance-Derived Heart Rate and Heart Rate Variability from tDCS Output Voltage: Sensorless Physiological Monitoring During Electrical Neuromodulation

**DOI:** 10.64898/2026.07.29.741585

**Published:** 2026-07-30

**Authors:** Yahia Abdalla, Benjamin Babaev, Kevin Walsh, Catarina Ferraz, Leigh Charvet, Giuseppina Pilloni, Marom Bikson, Mohamad FallahRad

## Abstract

**Background:**

Transcranial direct current stimulation (tDCS) devices adjust output voltage to maintain the target current despite varying impedance. Pulsatile blood flow produces beat-synchronous changes in tissue impedance.

**Objective:**

To determine whether impedance-derived heart rate (IHR), heart rate variability (IHRV), and respiration (IDR) can be estimated from tDCS output voltage without additional physiological sensors.

**Methods:**

A custom analog front-end acquires the tDCS output voltage across its full dynamic DC range and superimposed AC fluctuations with high precision. Beats detected from the AC-coupled signal yielded normal-to-normal intervals for HR, HRV, and interval-derived respiration. Accuracy was quantified as mean absolute error (MAE) in 10 healthy laboratory participants against ECG and respiration-monitor references, and against chest-strap RR intervals in 19 at-home sessions from 10 participants with mild-to-moderate depression.

**Results:**

Laboratory MAEs versus ECG were 0.57 bpm for HR, 9.40 ms for SDNN, and 18.90 ms for RMSSD (r = 0.995, 0.859, and 0.778; N = 10); respiratory-rate MAE was 1.36 breaths/min (r = 0.852; N = 6). Across 1-5 mA of tDCS, the cardiac voltage ΔV _cardiac_ (t) amplitude scaled linearly with current (slope, 0.073 mV/mA; p < 0.001). The pulsatile impedance ΔZ _cardiac_ (t) = (ΔV _cardiac_ (t)/I _applied_ ) amplitude averaged 0.080 ± 0.029 Ω (mean ± SD) across 50 participant-current observations, with no significant dependence on intensity (slope, −0.002 Ω/mA; p = 0.086). At-home MAEs were 1.43 bpm for HR, 8.86 ms for SDNN, and 24.92 ms for RMSSD (r = 0.995, 0.767, and 0.680; 19 sessions).

**Conclusions:**

tDCS output voltage contains a recoverable cardiac-synchronous signal arising from pulsatile impedance, enabling HR, HRV, and respiratory monitoring without additional physiological sensors.

## Full Text Availability

The license terms selected by the author(s) for this preprint version do not permit archiving in PMC. The full text is available from the preprint server.

